# Relative Power of Specific EEG Bands and Their Ratios during Neurofeedback Training in Children with Autism Spectrum Disorder

**DOI:** 10.3389/fnhum.2015.00723

**Published:** 2016-01-14

**Authors:** Yao Wang, Estate M. Sokhadze, Ayman S. El-Baz, Xiaoli Li, Lonnie Sears, Manuel F. Casanova, Allan Tasman

**Affiliations:** ^1^State Key Laboratory of Cognitive Neuroscience and Learning & IDG/McGovern Institute for Brain Research, Beijing Normal UniversityBeijing, China; ^2^Center for Collaboration and Innovation in Brain and Learning Sciences, Beijing Normal UniversityBeijing, China; ^3^Department of Bioengineering, J.B Speed School of Engineering, University of LouisvilleLouisville, KY, USA; ^4^Greenville Health System, Departments of Pediatrics and Biomedical Sciences, University of South Carolina School of Medicine GreenvilleGreenville, SC, USA; ^5^Department of Psychiatry and Behavioral Sciences, University of Louisville School of MedicineLouisville, KY, USA; ^6^Department of Pediatrics, University of Louisville School of MedicineLouisville, KY, USA

**Keywords:** electroencephalography, neurofeedback, autism spectrum disorder, gamma activity, EEG bands' ratios

## Abstract

Neurofeedback is a mode of treatment that is potentially useful for improving self-regulation skills in persons with autism spectrum disorder. We proposed that operant conditioning of EEG in neurofeedback mode can be accompanied by changes in the relative power of EEG bands. However, the details on the change of the relative power of EEG bands during neurofeedback training course in autism are not yet well explored. In this study, we analyzed the EEG recordings of children diagnosed with autism and enrolled in a prefrontal neurofeedback treatment course. The protocol used in this training was aimed at increasing the ability to focus attention, and the procedure represented the wide band EEG amplitude suppression training along with upregulation of the relative power of gamma activity. Quantitative EEG analysis was completed for each session of neurofeedback using wavelet transform to determine the relative power of gamma and theta/beta ratio, and further to detect the statistical changes within and between sessions. We found a linear decrease of theta/beta ratio and a liner increase of relative power of gamma activity over 18 weekly sessions of neurofeedback in 18 high functioning children with autism. The study indicates that neurofeedback is an effective method for altering EEG characteristics associated with the autism spectrum disorder. Also, it provides information about specific changes of EEG activities and details the correlation between changes of EEG and neurofeedback indexes during the course of neurofeedback. This pilot study contributes to the development of more effective approaches to EEG data analysis during prefrontal neurofeedback training in autism.

## Introduction

Informed clinical consensus defines autism as a behavioral syndrome characterized by pervasive impairments in several areas of development including social interaction, communication skills, and stereotypical interests and activities (American Psychiatric Association, 2013). Thus, far, there have been no neuropathological findings nor laboratory/performance based measures providing construct validity to the diagnosis. In the absence of pathognomonic abnormalities, clinical research in autism has been guided by a variety of ideologies and epistemological assumptions each contributing to the development of explanatory models or theories: executive function, weak central coherence, complex information processing, theory-of-mind, empathy, anatomical, and functional connectivity, etc. (Baron-Cohen, 1989; Frith and Happé, 1994; Ozonoff et al., 1994; Minshew et al., 1997; Ozonoff, 1997; Belmonte and Yurgelun-Todd, 2003; Belmonte et al., 2004; Baron-Cohen and Belmonte, 2005; Happé and Frith, 2006).

Recent studies by our group have characterized the neuropathology of autism as that of a minicolumnopathy (Casanova et al., 2002a,b, 2003, 2006a,b). Deficits within the inhibitory elements that surround the cell minicolumn suggest a mechanistic explanation to the cortical inhibitory/excitatory (I/E) imbalance in autism (Casanova et al., 2002a,b, 2013; Rubenstein and Merzenich, 2003). Oscillations and synchronization of pyramidal cells in and across minicolumns are maintained by networks of inhibitory GABAergic interneurons (Mann and Paulsen, 2007; Donner and Siegel, 2011). Local I/E interactions shape neuronal representations of sensory, motor, and cognitive variables, and produce local electroencephalographic (EEG) gamma frequency (30–80 Hz) oscillations. The I/E bias caused by faulty pyramidal cell-interneuronal dyads provides a receptive scenario to gamma frequency abnormalities in autism, and can be considered as a neurophysiological, EEG-based biomarker of autism. To the authors' knowledge every study on gamma frequencies in autism has shown abnormalities (Brock et al., 2002; Brown et al., 2005; Pavlova et al., 2006; Orekhova et al., 2007; Rippon et al., 2007; Baruth et al., 2010; Gross et al., 2012; Casanova et al., 2013; Sokhadze et al., 2014).

Strong evidence both from animals and human experiments indicated that high frequency gamma band oscillations, especially those around 40 Hz frequencies, are most directly associated with entrainment of local networks. Some experimental studies have found that 40 Hz centered gamma activity may correlate with human perceptual binding (Herrman and Knight, 2001) and attention (Bird et al., 1978). This is especially true for the 35–45 Hz range gamma sub-band in EEG which was thought to be closely associated with the mechanisms of information processing such as sensory, working memory, attention, and other cognitive processes (Tallon-Baudry et al., 1996; Tallon-Baudry and Bertrand, 1999; Herrman and Mecklinger, 2000; Grice et al., 2001; Gruber et al., 2001; Tallon-Baudry, 2003). Based on the above, abnormalities in the 35–45 Hz gamma oscillatory activity probably can be considered as one of the underlying causes of the cognitive deficits observed in autism.

Neurofeedback (NFB) has been recognized as a suitable tool for detecting and modulating neural plasticity due to its ability to non-invasively alter the excitability of neural circuits and for inducing a short-term functional reorganization in anatomically and functionally associated cortical and sub-cortical neural networks in the human cerebral cortex. By operant conditioning of EEG, NFB provides an effective way to train electrophysiological activity of the targeted cortical area. Multiple NFB studies indicate its usefulness as an efficacious neurotherapy for various mental disorders. Neurofeedback training is considered as one of the most effective and salient treatments for children with attention deficit/hyperactivity disorder (ADHD). The clinical efficacy of using NFB for ADHD treatments was supported by several meta-analyses of randomized clinical trials recently conducted (Lubar, 2003, 2004; Arns et al., 2009; Gevensleben et al., 2009; Sokhadze et al., 2009; Lofthouse et al., 2010). Since many autistic children also show signs of attention-deficit and hyperactivity some attempts have been made to use this technique as a treatment modality for ASD (Linden et al., 1996; Coben and Padolsky, 2007; Coben, 2008, 2013; Kouijzer et al., 2009a,b, 2010; Coben and Myers, 2010; Coben et al., 2010; Sherlin et al., 2010; Thompson et al., 2010a,b; Linden and Gunkelman, 2013). Several current papers review the use of neurofeedback for ASD treatment and many of them provide evidence that some of the core symptoms of autism can be improved this way (Jarusiewicz, 2002; Coben and Padolsky, 2007; Coben, 2008, 2013; Kouijzer et al., 2009a,b; Coben and Myers, 2010; Coben et al., 2010; Sokhadze et al., 2014). During NFB procedure, subjects are trained to enhance desired electro-cortical activity, while suppressing undesirable activity. Through the NFB training course many symptoms related to EEG abnormalities can be corrected and improved toward normalization.

Neurofeedback is relatively new form of treatment for ASD (Kouijzer et al., 2010). There were several case, pilot and group studies (Sichel et al., 1995; Jarusiewicz, 2002) followed by controlled group studies (Coben and Padolsky, 2007; Kouijzer et al., 2009a,b; Coben and Myers, 2010). More detailed accounts summarizing behavioral, cognitive, and neurophysiological data can be found in current reviews (Thompson et al., 2010a,b; Coben, 2013; Linden and Gunkelman, 2013). Among controlled studies should be specifically mentioned quantitative EEG (qEEG) and connectivity analysis guided studies conducted by Coben and his associate (Coben and Padolsky, 2007; Coben and Myers, 2010; Coben et al., 2010; Coben, 2013). QEEG based assessment of functional connectivity is proposed to guide neurofeedback intervention in autism. Some researchers use qEEG-based subtypes or so called endophenotypes to guide neurofeedback in ASD (Linden and Gunkelman, 2013). These techniques use individualized approaches to selection of neurofeedback-based treatment in autism.

QEEG assessments (so called “brain mapping”) and training using multiple EEG sensors (usually > 19) is necessary for qEEG-guided neurofeedback. The analysis of current trends in neurofeedback application in ASD shows that multichannel recordings appear to be the way the field is moving for more advanced training models, though for some applications fewer channels are required and seem to be sufficient for positive outcomes. In hyperactive individuals, or in a sensory over-sensitive autistic child using an EEG cap and preparing skin with abrasive and electrolyte gels may be less feasible. Also some individuals who could really benefit from multichannel EEG recordings and qEEG-guided neurofeedback may not be able to tolerate the EEG caps application and gel in procedures that are used now. Relatively simple to apply one or two-channel wireless neurofeedback systems have certain advantages for neurofeedback training in children with autism.

In the present study we planned to develop methodology to monitor EEG activity and analyze changes during neurofeedback sessions in high-functioning children with ASD. The study represents one of approaches aimed at the understanding of EEG correlates of neurofeedback training in high functioning ASD population, rather than an attempt at claiming clinical improvements resulting from the prefrontal brainwave training. More research studies should be done to understand: (1) whether children with high functioning autism can control EEG in NFB mode, (2) how EEG characteristics are changing during the training course in an ASD population, and (3) what additional efforts are needed to correctly identify specific changes in EEG rhythms known to be abnormal in ASD, specifically gamma activity at the frontal sites.

Our approach included neurofeedback training at the prefrontal topography, specifically at the midline prefrontal site. Considering the role of the prefrontal cortex in executive functions, including attention and cognitive processes, it was feasible to investigate effects of neurofeedback using training at the anterior, frontal location rather than at the central, or posterior (e.g., parietal) sites. This selection of cortical topography was also determined by our prior studies on gamma oscillations in children with autism (Sokhadze et al., 2009; Baruth et al., 2010; Sokhadze, 2012; Casanova et al., 2013) that showed alterations of evoked and induced gamma oscillations during attention tests especially well present at the frontal topographies.

The goal of this study was to conduct neurofeedback in children with ASD using the PAT neurofeedback device with the “Focus/Neureka!” (“ Focused Attention” index and “40 Hz-centered Gamma” index) training protocol of Peak Achievement Trainer (PAT) neurofeedback device (Neurotek, Goshen, KY) to investigate: relative changes in EEG bands (e.g., theta [4–8 Hz]) and sub-bands of interest (e.g., low beta [13–18 Hz], high beta [18–30 Hz]) and their ratios (e.g., theta/low beta, etc.) throughout the entire 18 session long course of neurofeedback training in ASD, and during each individual training session using custom-made Matlab application, how gamma power and EEG bands power ratios are changing during individual sessions and between sessions within the course of neurofeedback training in high functioning individuals with ASD, and whether there are any correlation between EEG measures of interest (i.e., relative gamma power, theta/beta ratio) and neurofeedback training indices such as “Focused Attention” index and “40 Hz-centered Gamma” index (Cowan and Albers, 2008).

It was expected that all participants would complete 18 weekly sessions of ~25–30 min long training and learn to increase the “Focused Attention” measure, and control level of “40 Hz centered Gamma” parameter in neurofeedback mode. It was similarly expected that an increase in so called “Focused Attention” measure of the PAT device protocol would be manifested in a gradual decrease of theta/low beta and theta/high beta EEG ratios, while an increase in the “40 Hz centered Gamma” measure would be accompanied by the gradual increase of the relative power of gamma (30–45 Hz) band.

## Methods

### Patient demographics and recruitment

Eighteen children and adolescents with ASD (mean age 13.2 years, SD = 4.3, 4 females, 14 males) were recruited in this study. It was not required for participants to be off medication during the whole course of the neurofeedback trainings. Medication status, dosage, and other variables of pharmacotherapy were accurately monitored and recorded, but were not used as a part of the patients' demographic descriptive characteristics in this study. Before the neurofeedback session, on the days of their visit, all participants were requested not to take medication. Participants with ASD were recruited through the University of Louisville Weisskopf Child Evaluation Center (WCEC). All participants with ASD were diagnosed by an experienced pediatrician according to the Diagnostic and Statistical Manual of Mental Disorders (DSM-IV-TR) (American Psychiatric Association, 2000) and further ascertained with the Autism Diagnostic Interview—Revised (ADI-R, Le Couteur et al., 2003). Further, medical estimations were made to exclude the participants with a history of seizure, significant hearing, or visual impairment, a brain abnormality or an identified genetic disorder. Participants with severe psychiatric comorbidities were not included in the study. All patients were naive to neurofeedback training procedures and never participated in any neurofeedback study before.

Using the Wechsler Intelligence Scale for Children (WISC-IV, Wechsler, 2004) or (for adolescents) the Wechsler Abbreviated Scale of Intelligence (WASI) (Wechsler, 1999), all participants were assessed to have full-scale IQ > 80. Fourteen participants were high-functioning persons with autism diagnosis and four had Asperger Syndrome. Child and adolescent psychiatrist and clinical psychologist at the WCEC performed pre- and post-neurofeedback clinical evaluations. Neurofeedback sessions were conducted by an experienced applied psychophysiologist. All required IRB-approved consent/assent forms were signed by the participants and their parents/guardians.

### Behavioral measures and evaluation

In conjunction with the EEG data we collected the behavioral rating results with the pre- and post-neurofeedback data using the Aberrant Behavior Checklist (ABC) (Aman and Singh, 1994) from the parents of the ASD participants. *Irritability, Lethargy/Social Withdrawal, Stereotypy, Hyperactivity*, and *Inappropriate Speech* were the five problem aspects that were contained in and assessed by the ABC rating scale. In the current study, we focus on the *Hyperactivity, Lethargy*, and *Irritability* ratings before and after a course of NFB treatments.

### Neurofeedback protocol and data collection

In the study, ASD participants completed a course of NFB trainings using a “Focus/Neureka!” protocol of the PAT neurofeedback device designed to modulate the “Focused Attention” index (FAI) and “40 Hz-centered Gamma” index (GPI). The prefrontal neurofeedback training protocol used in this study was based on the BioExplorer software (CyberEvolution, Seattle, WA, USA) platform. The protocol provided the exercises for each subject to enhance the single-pointed “Focused Attention” index measure (FAI) throughout the session while maintaining an adequate level of “Neureka!” measure (GPI) within a certain range. During all of the treatment sessions different scenes from the BBC “Planet Earth” and “Life” series were shown to maintain the participants' adherence. The protocol in this study provides feedback to the subjects in both visual and auditory modalities. Based on the thresholds set, parameters related with visual feedback such as the brightness, size, and continuation of the video have been modulated and the sound volume of the video adjusted simultaneously according to the “FAI” and “GPI” measures during the treatment. All EEG signals and training parameters were measured using three electrodes, one active electrode at the prefrontal EEG (FPz) site, the second being a reference on the left ear, and a third sensor serving as ground and located between the two above electrodes. All of the subjects in the study were requested to complete a 25–30 min recording per session and a total of 18 weekly neurofeedback sessions, in order to increase the “FAI” and “GPI” using the “Focus/Neureka!” PAT protocol. More than 90% of the sessions met the requirement of a 20-min minimum usable EEG data recording. Eye blink and EMG artifacts removal was implemented using the specific BioExplorer application that can be found in the operation manual of the NFB device.

### The EEG signal processing

The EEG signal collected and recorded by BioExplorer applications during NFB treatments were exported and further analyzed by a series of customized codes using Matlab software (MathWorks, Inc, Massachusetts). As an extension application of BioExplorer software, BioReview report can be called to export the raw EEG and the desired frequency bands of data for each session. By configurations in the BioReview report, along with the raw EEG, the separated delta (2–4 Hz), theta (4–8 Hz), alpha (8–13 Hz), low beta (13–18 Hz), high beta (18–30 Hz), and gamma (30–45 Hz) were also acquired using 7th order elliptical bandpass filters. The exported data have been arranged in a text file in which the different items were organized into columns and each subsequent row represented the data point in time series between samples.

For the relative power calculation, it was necessary to gain the total power of the band from 2 to 45 Hz (the whole bands from delta to gamma frequencies). A custom band-pass filter application integrated of wavelet transformation and a Harris window configuration were created to filter and separate the 2–45Hz frequency band from the raw signal that was exported from the BioReview reports. The wavelet analysis was used to provide enhanced temporal resolution of frequency responses of a given signal and it allowed us to apply a band pass filter to the individual waveform and avoid the distortion when applying the filter to the entire signal. In the study, the sample-rate of the raw signals collected in the Bioexplorer system was 256 Hz. The EEG changes in the prefrontal site during each session for all subjects (20 min data per session and 18 sessions for each subject) were analyzed in Matlab. The continuous wavelet transformation of the signal is shown in the Equation below, in which

S(scale) = 1/ *frequency*; τ = time shift; Psi(ψ) = mother wavelet(in our case the Morlet window).

$$\begin{array}{l} {CWT}_{\chi }^{\psi }\left(\tau ,s\right)={\text{Ψ}}_{\chi }^{\psi }\left(\tau ,s\right)=\frac{1}{\sqrt{S}}\int \chi \left(t\right)\psi^{*}\left(\frac{t-\tau }{s}\right)dt \end{array}$$

In our codes, the Morlet window was used to separate the raw signal (the first column in the text file exported from the Bioexplorer) into 128 coefficients. And the coefficients then were filtered into 2–45 Hz frequency by applying the Harris window.

The equation for the 4-term Harris window of length N is shown below.

$$\begin{array}{l} \omega \left(\text{n}\right)=a_{0}-a_{1}\cos\left(\frac{2\pi n}{N-1}\right)+a_{2}\cos\left(\frac{4\pi n}{N-1}\right)-a_{3}\cos\left(\frac{6\pi n}{N-1}\right)\\ a_{0}=0.355768;a_{1}=0.487396;a_{2}=0.144232;a_{3}=0.012604; \end{array}$$

**Figure 1:**
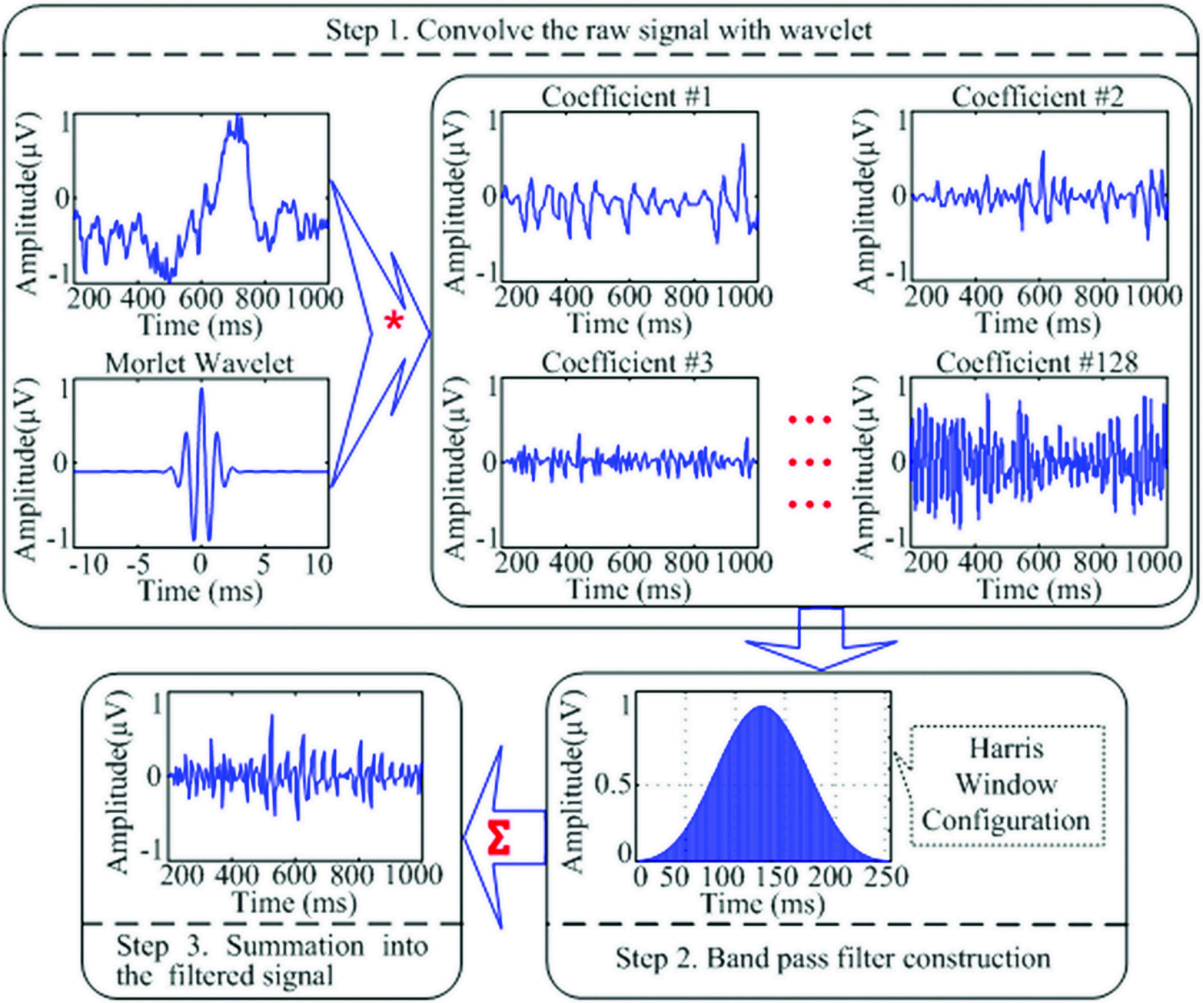
**A schematic representation of the wavelet transformation and band-pass filtering applications utilized to filter raw EEG signal into the desired filtered signal to be used for relative power calculation of the EEG bands of interest**. “^*^” Means the process of “convolution”; “∑” means the process of “summation”; the three lines of “…” are the ellipsis points, which means in the similar fashion from “Coefficient #3” to “Coefficient #128.”

The Harris window is employed for convolution with each coefficient. It provides a good method to avoid the leakage of the high variable dynamic EEG signals. At last, the filtered coefficients were transformed and summed to produce a reconstructed filtered signal (Figure 1).

Besides the relative power calculation for each band, the ratio of certain bands was also calculated. The formula used to calculate relative power is given in the equation below, where B represents the band's signal and T represents the total signal (2–45 Hz).

$$\sum_{0}^{i}{ℬ}^{2}/\sum_{0}^{i}T^{2}$$

The ratios of interest for this study were theta (4–8 Hz) to low beta (13–18 Hz)—Theta/low beta ratio, theta to high beta (18–30 Hz)—Theta/high beta ratio, and cumulative theta to beta (13–30 Hz) ratio (Theta/beta).

### Statistical analysis

The primary statistical analyses in the study mainly included linear regression estimation and paired sample *t*-test methods. Each EEG dependent variable over 18 sessions of neurofeedback course was analyzed using linear regression analyses and the mean values of dependent EEG variables at the first and last session of the NFB course together with the pre- and post-NFB behavioral measures using ABC questionnaire were compared with the paired sample *t*-test method. EEG variables and “FAI” and “GPI” NFB training indices were calculated as well on per minute basis during each training session. Each dependent EEG variable went through the normality distribution analysis using *t*-test to ensure appropriateness for the test, and 95% confidence intervals (95% CI) were included in outcomes. Pearson correlation analysis was used for individual EEG measures, neurofeedback training indices, and behavioral measures collected using ABC questionnaire.

## Results

### EEG activity measures across 18 sessions of neurofeedback

**Figure 2:**
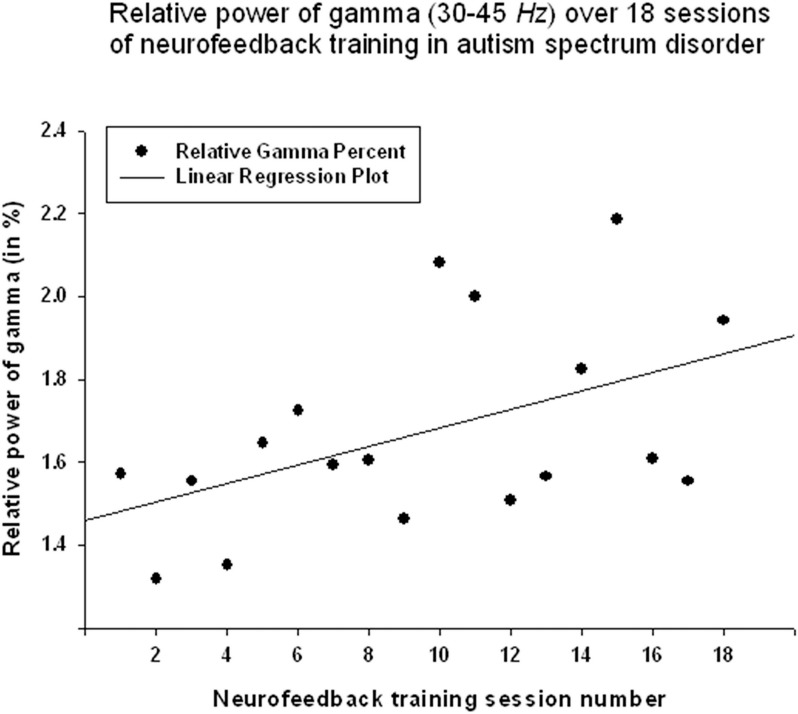
**Linear regression of the relative power of Gamma band over 18 sessions of neurofeedback training in 18 children with ASD (***R*** = 0.491, ***y*** = 0.022x + 1.45%, ***t*** = 2.25, ***p*** = 0.039)**.

Relative power of gamma activity (power within 35–45 Hz vs. total power in 2–45 Hz, in percentage) showed statistically significant linear increase over 18 sessions of neurofeedback (linear regression: R = 0.491, *R*^2^ = 0.241, *y* = 0.022x + 1.45%, *t* = 2.25, *p* = 0.039, power of test 0.55 at α = 0.05, below the desired level of 0.80, Figure 2, Table 1). However, paired sample Student's *t*-test showed that relative power of gamma did not increase statistically from the first to the last neurofeedback session [from 1.57 ± 1.30% to 1.80 ± 1.15%, mean increase 0.23 ± 0.29%, *t*_(17)_ = 0.76, *p* = 0.456, n.s., Table 2].

**Table 1:** **Summary of linear regression statistics for main dependent variables in 18 sessions of NFB**.

**Measures**	**Units**	***t***	***P*-value**	***R***	***R*^2^**	**Regression equation**	**Power**
Gamma	%	2.25	0.039	0.491	0.241	*y* = 0.022x + 1.45	0.548
Theta/low beta	N/A	−3.57	0.003	0.666	0.444	*y* = −0.079x + 9.49	0.876
Theta/high beta	N/A	−4.01	0.001	0.708	0.502	*y* = −0.088x + 6.26	0.928
“Focus Attention” index	C.U.	1.84	0.084	0.418	0.175	*y* = 0.056x + 73.83	0.408
“40 Hz Gamma” index	C.U.	2.61	0.019	0.547	0.299	*y* = 0.165x + 42.37	0.662

**Table 2:** **Paired sample ***t***-test of the last vs. first neurofeedback session in 18 subjects**.

**EEG measures Last-minus-first**	**Units**	**Paired differences**				***t***	**df**	***P*-value**
		**Mean**	**Std. Dev**.	**95% CI**				
				**Lower**	**Upper**			
Gamma	%	0.22	1.24	−0.84	0.39	0.76	17	0.456
Theta/low beta	N/A	−1.72	3.40	0.032	3.42	−2.15	17	0.046
Theta/high beta	N/A	−1.48	2.83	0.081	2.89	−2.23	17	0.039
Theta/beta	N/A	−1.26	2.47	0.033	2.49	−2.16	17	0.045
“Focused Attention” index	C.U.	2.29	2.25	−3.41	−1.17	4.32	17	0.001
“40 Hz-centered Gamma” index	C.U.	3.68	6.66	−7.00	−0.37	2.34	17	0.031

**Figure 3:**
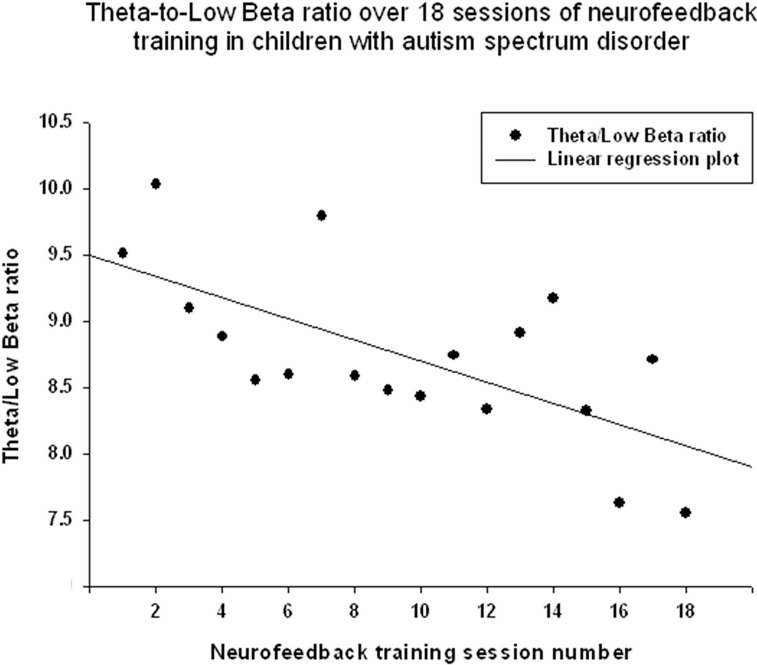
**Linear regression of the theta/low beta ratio over 18 sessions of neurofeedback training in 18 children with ASD (***R*** = 0.666, ***y*** = −0.079x + 9.49, ***t*** = −3.57, ***p*** = 0.003)**.

**Figure 4:**
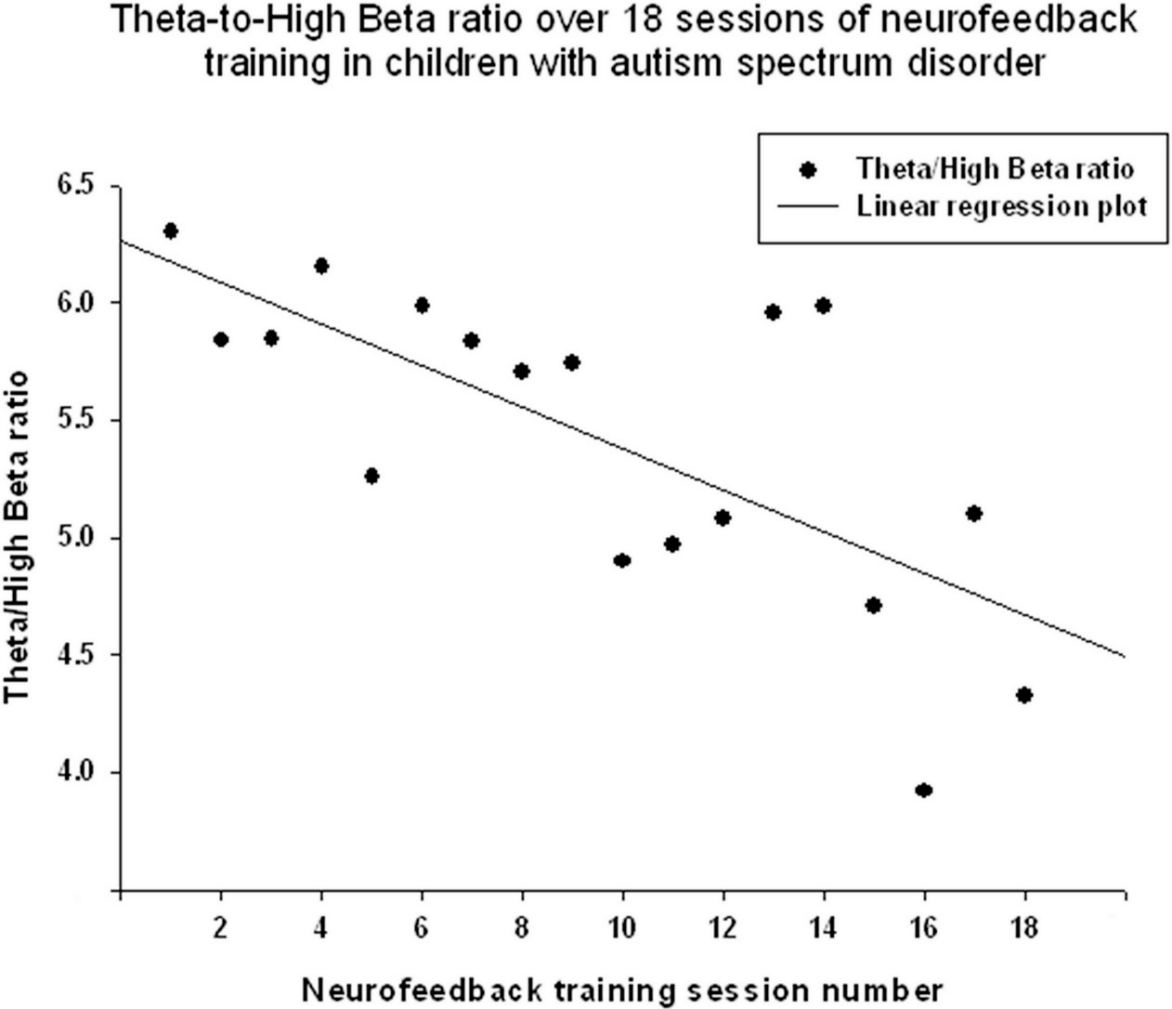
**Linear regression of the theta/high beta ratio over 18 sessions of neurofeedback training in 18 children with ASD [***R*** = 0.708, ***y*** = −0.088x + 6.24, ***t***_**(17)**_ = −4.01, ***p*** = 0.001]**.

Theta/low beta ratio showed a statistically significant linear decrease over 18 sessions of neurofeedback (R = 0.666, R^2^ = 0.444, *y* = −0.079x + 9.49, *t* = −3.57, *p* = 0.003, power = 0.87, Figure 3) and *t*-test showed that theta/low beta ratio decreased statistically from the first to the last neurofeedback session [from 9.54 ± 3.57 to 7.81 ±1.46, mean decrease being −1.72 ± 3.40, *t*_(17)_ = −2.15, *p* = 0.046]. Regression of the theta/high beta ratio over 18 sessions showed a significant linear correlation [R = 0.708, *R*^2^ = 0.502, *y* = −0.088x + 6.24, *t*_(17)_ = −4.01, *p* = 0.001, power = 0.92, Figure 4]. *T*-test showed a significant decrease from the first to the last session [from 6.22 ± 3.11 to 4.73 ± 2.16, *t*_(17)_ = −2.23, *p* = 0.039]. The ratio of theta/beta (i.e., theta ratio to sum of low and high beta, 13–30 Hz) showed a similar decrease trend [−1.26±2.47, *t*_(17)_ = −2.16, *p* = 0.045].

### Neurofeedback training indices

**Figure 5:**
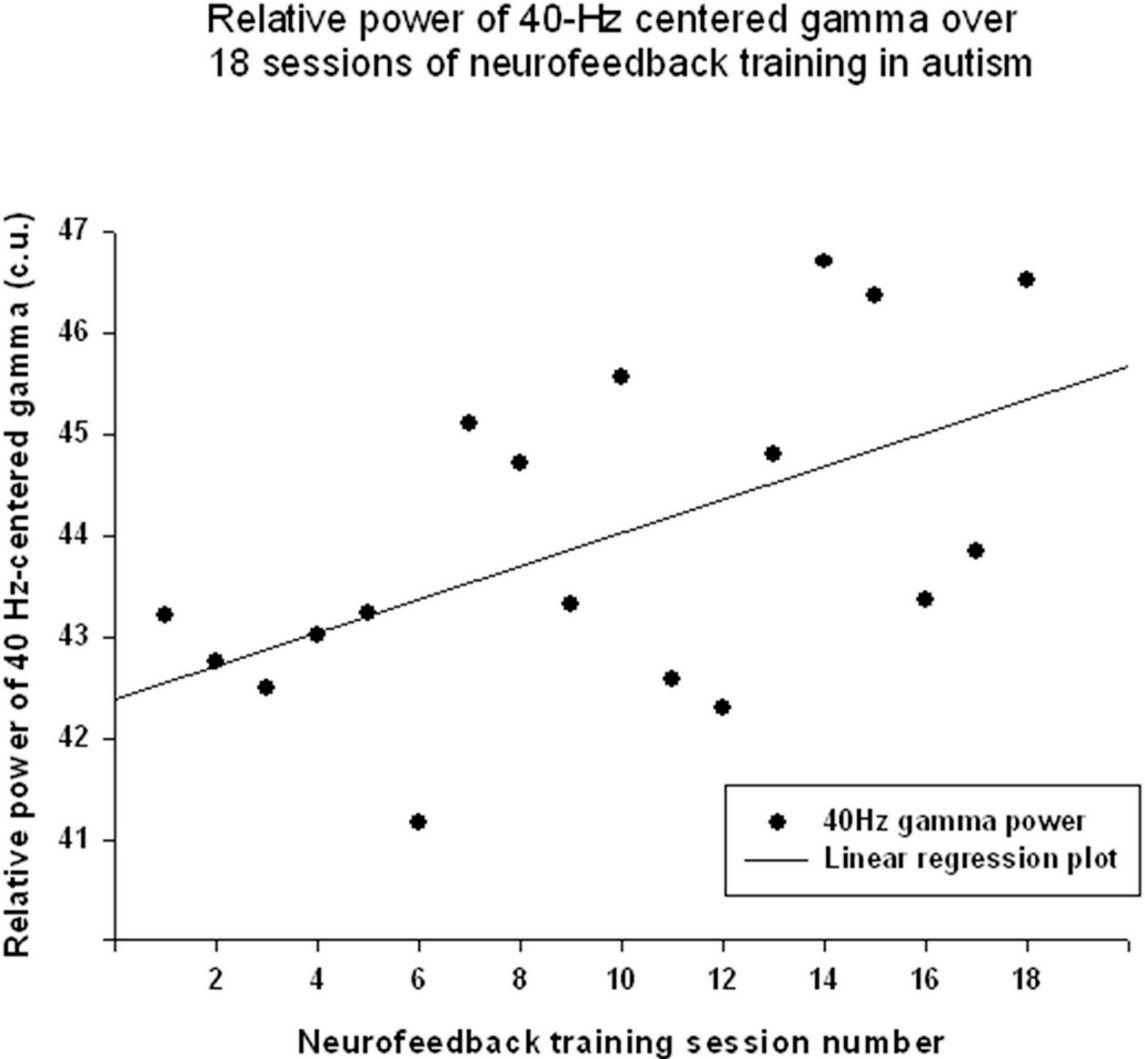
**Linear regression of the “40-Hz centered Gamma” index over 18 sessions of training [***R*** = 0.547, ***y*** = 0.165x + 42.37, ***t***_**(17)**_ = 2.61, ***p*** = 0.019]**.

The “Focused Attention” index (FAI, i.e., “Inhibit All” measure in neurofeedback) did not show a statistically significant linear increase over 18 sessions of training [R = 0.418, *R*^2^ = 0.175, *t*_(18)_ = 1.84, *p* = 0.084, n.s.], but *t*-test showed significant changes from the first to the last session of neurofeedback [from 73.1 ± 4.85 to 75.39 ± 5.27 c.u., *t*_(17)_ = 4.32, *p* = 0.001]. The other neurofeedback measure reflecting relative power of “40-Hz centered Gamma” index did show a linear increase trend over 18 sessions of training [R = 0.547, R^2^ = 0.299, *t*_(17)_ = 2.61, *p* = 0.019, power = 0.66, Figure 5] and paired sample *t*-test confirmed that the change of this index from the first to the last session was statistically significant [from 42.34 ± 7.45 to 46.03 ± 6.13 c.u., *t*_(17)_ = 2.34, *p* = 0.031]. This neurofeedback index showed a significant positive Pearson correlation coefficient with relative gamma power across 18 session of training (*r* = 0.548, *p* = 0.019). On the other hand, the “Focused Attention” index showed a negative correlation with the theta/low beta ratio (*r* = −0.51, *p* = 0.03) and with the theta/beta ratio (*r* = −0.59, *p* = 0.01) across 18 sessions of neurofeedback.

### EEG and neurofeedback training measures during 20 min session

**Figure 6:**
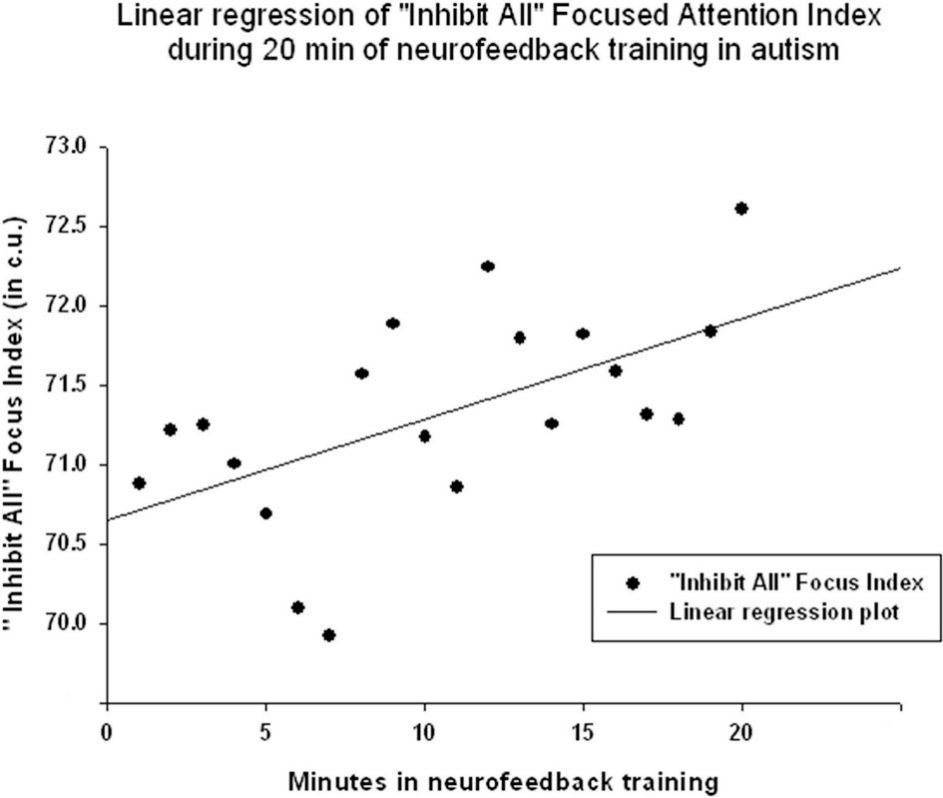
**Linear regression of the “Focused Attention” index during 20 min long neurofeedback session (mean for all subjects across all sessions [***R*** = 0.576, ***y*** = 0.063x + 70.64 c.u., ***t***_**(19)**_ = 2.99, ***p*** = 0.008]**.

**Figure 7:**
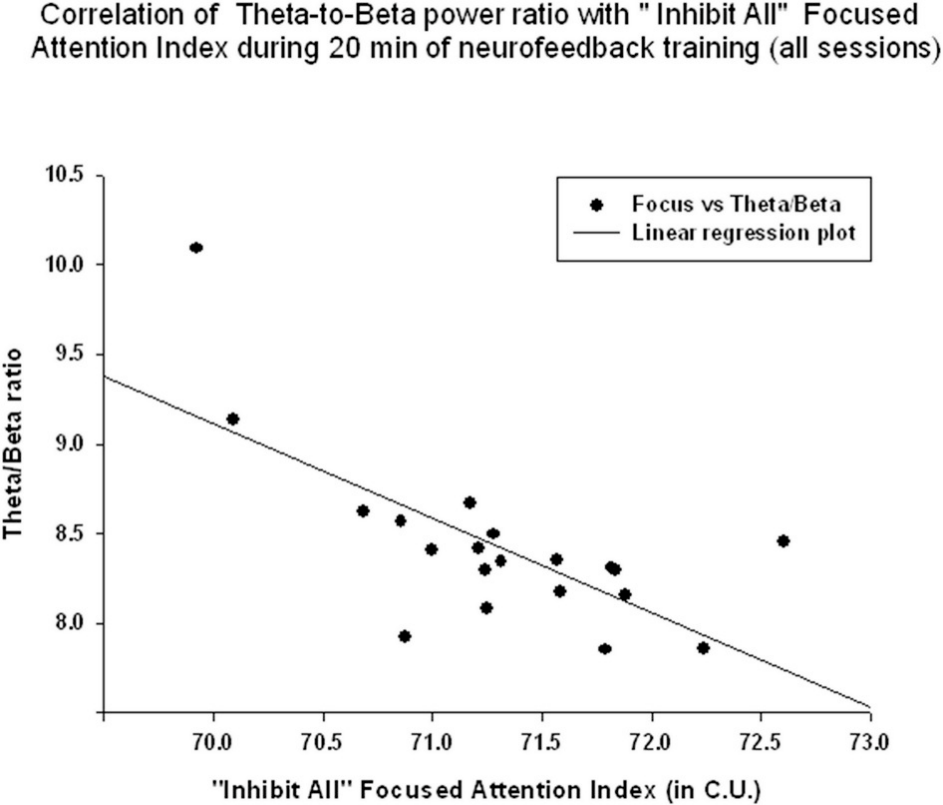
**Correlation of the theta/beta with the “Focused Attention” index during 20 min of neurofeedback training (across all sessions, ***r*** = −0.70, ***p*** = 0.001)**.

EEG measures did not show a significant linear regression over 20 min of neurofeedback session. The “Focused Attention” index did show a statistically significant linear increase during each neurofeedback session [R = 0.576, R^2^ = 0.332, *y* = 0.063x + 70.64 c.u., *t*_(19)_ = 2.99, *p* = 0.008, power = 0.77 at α = 0.05, Figure 6]. This index had a significant negative correlation with the theta/beta ratio during 20 min long neurofeedback sessions (*r* = −0.70, *p* = 0.001, Figure 7). It should be noted that the theta/low beta and theta/high beta ratios showed a high level positive correlation during training sessions (*r* = 0.63, *p* = 0.003).

### Behavioral evaluations

There was a significant reduction in *Lethargy/Social Withdrawal* subscale of the ABC. The rating scores showed a reduction [from 10.18 ± 6.07 to 7.53 ± 5.82, change was −2.64 ± 3.13, *t*_(17)_ = 3.29, *p* = 0.005], while *Hyperactivity* scores also showed decrease [from 16.65 ± 13.78 to 13.29 ± 11.97, −3.35±5.39, *t*_(17)_ = 2.56, *p* = 0.021].

Changes in *Lethargy/Social Withdrawal* scores showed a positive correlation with relative gamma power changes (*r* = 0.43, *p* = 0.041), and negative correlation with the theta/low beta (*r* = −0.43, *p* = 0.043) and theta/high beta ratios (*r* = −0.45, *p* = 0.033). *Hyperactivity* scores on ABC did not show any statistically significant correlation with EEG measures (i.e., theta/beta ratio, gamma) or NFB indices (i.e., “Focus” index, “40 Hz Gamma” index). Changes on other rating scales of the ABC did not reach a significance level. For instance, *Irritability* rating showed a trend to decrease from 9.59 ± 7.65 to 8.17 ± 7.13, changing by −1.41±4.33, *t*_(17)_ = 1.35, *p* = 0.198, n.s. Change of *Stereotypy* was only −0.88±2.97, *t*_(17)_ = 1.22, *p* = 0.239. Changes in *Inappropriate Speech* scores were even smaller (−0.29±1.86, *p* = 0.524).

## Discussion

The results indicate that the study outcomes were very close to the predicted ones, especially in regards to changes in the ratios of interest (theta/low beta, theta/high beta, theta/Beta) from the first to the last session, and regression of the dependent variables across the neurofeedback training sessions. For instance, the theta/beta showed a decrement across NFB sessions (Figure 3) while the relative power of the gamma band showed a linear increase over the course of the training (Figure 2). Both neurofeedback training indices (“Focused Attention” index and “40 Hz Gamma” index) showed a linear increase over training sessions and increased significantly toward the end of the course. We could not find, however, significant trends of the EEG variables changes within the 20 min of individual neurofeedback sessions. Only one training index (i.e., “Focused Attention,” Figure 6) showed a linear increase over the minutes within individual sessions and high negative correlation with both theta/low beta and theta/high beta ratios in the EEG (Figure 7).

We found a notable decrease in the theta/low beta and theta/high beta proportions from session to session along with an increase of both training indices to the end of the course. These results are in accordance with the goals of NFB treatment described earlier for children with ADHD (Arns et al., 2009; Lofthouse et al., 2010). Even though the theta/low beta ratios used in prior ADHD NFB studies were mostly collected from the central cortical sites (e.g., Cz), our frontal theta/low beta ratios showed similar trends in ADHD population (Hillard et al., 2013; Sokhadze et al., 2013). In this study, a reduction in the theta/beta proportions at the prefrontal site was robust across sessions. Due to the improvements in behavioral outcomes indicated by ABC questionnaire, it is also possible to discuss whether training of the “Focus/40 Hz Gamma” measures of the PAT protocol are related to functional behavioral improvements reported by the patients. Determining which of these two measures is more fundamental to the effects of neurofeedback in ASD would increase the efficiency and aid in the delivery of more effective neurofeedback treatment methods.

As mentioned earlier, autism is characterized by an imbalanced inhibitory/excitatory ratio in local cortical network, which may cause the disordered gamma oscillations in ASD reflected at the electroencephalographic level. The gamma abnormalities and excessive cortical excitation (E/I ratio) in autism have been considered as an important EEG biomarkers for ASD based on recent theoretical reviews and experimental studies (Casanova et al., 2015; Uzunova et al., 2015). Brown et al. (2005) interpreted the abnormal gamma responses in their study on individuals with autism as reflecting decreased “signal to noise” ratio due to decreased inhibitory processing (Grice et al., 2001; Lansbergen et al., 2011). Brock et al. (2002) described the parallels between the psychological model of “central coherence” (Frith and Happé, 1994) in information processing and their neuroscience model of neural integration or “temporal binding” (Szentagothai and Arbib, 1975). This concept was further elaborated in an “impaired connectivity” hypothesis of autism which summarized theoretical and empirical advances in research implicating disordered connectivity in autism (Brown, 2005). The authors highlighted recent developments in the analysis of the temporal binding of information and the relevance of gamma activity to current models of structural and effective connectivity based on the balance between excitatory and inhibitory cortical activity (Casanova et al., 2002b, 2013; Belmonte and Yurgelun-Todd, 2003; Belmonte et al., 2004; Rippon et al., 2007). Based on the minicolumn hypothesis of autism, disrupted patterns of coordinated high frequency oscillatory output in distributed networks might be associated with cortical “disconnection” in autism according to Casanova et al. (2006a,b).

The current study indicated the effectiveness of prefrontal neurofeedback aimed at modulating the disordered EEG activities associated with ASD. Also, from the results of the correlation between “40 Hz Gamma” index and the relative power of gamma calculated in our custom made program, the “Focus/40 Hz Gamma” protocol provided by the neurofeedback equipment used in our study can effectively help to improve gamma activity along with the decrement of theta/beta ratio in prefrontal EEG in children with ASD (Sokhadze et al., 2009; Casanova et al., 2015). Further, the results of the relative power of gamma and the band ratios that were calculated in our wavelet transform based program show us more details of how ASD subjects were controlling their EEG during the NFB treatments. Exported from the NFB software, the raw EEG data were filtered and calculated in our own program developed in the study. The Wavelet transformation based program could provide us the enhanced temporal resolution of frequency responses of a given signal and help to acquire the accurate and intact dynamic information. As a non-linear time-varying signal, EEG frequency data are suitable for being analyzed by Wavelet transformation algorithms. The custom made codes developed in the study provide us an important off-line method for clearly detecting the specific changing characteristics of EEG activities during the NFB treatments.

Theta/beta ratios (both theta/low beta and theta/high beta) showed the significant linear decrease over 18 sessions of neurofeedback, and in addition *t*-test showed that the ratios decreased statistically from the first to the last NFB training sessions. Theta/beta ratio is one of the classical indices for characterizing the ability to focus attention and to concentrate. The current study showed that both prefrontal theta/beta ratio and power of gamma activity could be modulated positively by operant conditioning during the NFB training in high functioning children with ASD. It is well-known that most ASD subjects have difficulties with switching focused attention. The “Focus/40 Hz Gamma” protocol used in the study provided a successful way for positively modulating both gamma activity and focused concentration abilities in ASD. The positive effects of the neurofeedback training further can be manifested by the improvement in the behavioral scores measured by the ABC. Our results show a significant reduction in the *Lethargy/Social Withdrawal* subscale of the ABC and a negative correlation with the theta/beta ratio. The *Hyperactivity* scores of ABC also showed a decrease but the same did not correlate with any EEG or NFB indices used in this study. The improvement of behavioral changes assessed by ABC before and after the 18 sessions of NFB treatments was in accordance to the functional outcomes seen in the EEG profile changes. Our study showed that compared to previous protocols that required more sessions per subject (> 30) and a more frequent training rate (e.g., twice per week), the statistical significant improvement either in EEG or in behavioral measures (Sokhadze et al., 2009) can also be achieved within a shorter number of sessions (i.e., 18 NFB sessions in ASD, or even 12 sessions in ADHD, Hillard et al., 2013) and weekly visits. Probably more than 18 sessions might contribute to better consolidation of results of operant conditioning using neurofeedback, and currently we have studies in progress that will compare outcomes of 12 vs. 18 vs. 24 sessions of neurofeedback using the same protocol in children with autism. Our future efforts will be directed to combine the neurofeedback with other novel neuromodulation techniques employed in autism treatment (e.g., rTMS, tDCS, auditory integration training, etc.).

EEG oscillations in the gamma band, and specifically those centered around 40 Hz has been historically associated with feature binding and cognitive processes (Tallon-Baudry et al., 1996), and neurofeedback studies targeted at changing 40 Hz gamma power popular back in 70s and 80s (Bird et al., 1978) are still attracting interest of investigators (Keizer et al., 2010). One of the important characteristics of our version of 40 Hz centered gamma power upregulation was integration of gamma training with the task of maintaining focused attention index. Another important feature of the protocol specifics was recording gamma activity at the midline prefrontal site. One more specific feature of the protocol used in this study was weekly training sessions that allowed subjects to stay longer in treatment. However, the major difference from other studies using neurofeedback in autism was in careful analysis of the dynamics of EEG bands of interest (i.e., theta, beta, and gamma) and their ratios during individual neurofeedback sessions and across the whole 18 sessions-long course of treatment. The study provided additional solution to analysis of EEG during neurofeedback training using custom-made Matlab application.

It should be noted that the study has several limitations. The enrollment to the neurofeedback training was open to only high-functioning children with autism and children with Asperger syndrome, thus results cannot be directly interpolated for low functioning children with ASD. The study was not designed as a clinical research as it had no control group of participants, and the number of clinical behavioral evaluations was minimal. The focus of current study was directed toward more accurate analysis of EEG signal using custom-made software with the aim of exploration of the dynamics of EEG activity during the neurofeedback training course in children with autism. Records of patients demographic specifics (e.g., social status of families, ASD onset, and duration data, etc.) and detail of their medication status were not analyzed.

In order to foster the neurofeedback treatment applications for children with ASD and its scientific rationale, further methodological advances are necessary: controlled and randomized study designs, larger sample sizes of patients, a more accurate selection of subjects with ASD, and more intensive and rigorous baseline, post-treatment- and follow-up evaluations.

## Author contributions

For the manuscript, YW is responsible for the data processing and writing; Prof. ES mainly focused on the experiments design and the whole procedures; Prof. AE developed the program in Matlab; Prof. XL help to review the Matlab codes; Dr. LS mainly responsible for the behavioral assessments for ASD subjects during the experiments; Prof. MC partially provided the financial supports for the whole study; Prof. AT reviewed the manuscript and did many modifications for it.

### Conflict of interest statement

The authors declare that the research was conducted in the absence of any commercial or financial relationships that could be construed as a potential conflict of interest.

## Abbreviations

ABC: Aberrant Behavior Checklist
ADI-R, Autism Diagnostic Inventory: Revised
ASD: autism spectrum disorder
ADHD: attention deficit hyperactivity disorder
EEG: Electroencephalogram
EMG: Electromyogram
FAI: Focused Attention index
GPI: Gamma power index (40 Hz centered gamma power)
NFB: neurofeedback
PAT: Peak Achievement Trainer
rTMS: repetitive Transcranial Magnetic Stimulation
tDCS: transcranial direct current stimulation
WCEC: Weisskopf Child Evaluation Center
WASI: Wechsler Abbreviated Scale of Intelligence
WISC: Wechsler Intelligence Scale for Children.
